# The Difference in Interleukin-19 Serum on Degrees of Acne Vulgaris Severity

**DOI:** 10.1155/2018/4141579

**Published:** 2018-04-01

**Authors:** Moerbono Mochtar, Alamanda Murasmita, M. Eko Irawanto, Indah Julianto, Harijono Kariosentono, Fajar Waskito

**Affiliations:** ^1^Dermatovenerology Department, Sebelas Maret University, Dr. Moewardi General Hospital, Surakarta, Indonesia; ^2^Dermatovenerology Department, Gadjah Mada University, Dr. Sardjito' General Hospital, Yogyakarta, Indonesia

## Abstract

**Introduction:**

Acne vulgaris is a multifactorial disease. Recent study showed that inflammation does have a central role in the formation of both inflammatory and noninflammatory lesions in acne vulgaris. There are various findings of proinflammatory cytokines related to acne vulgaris, but no previous study correlate interleukin- (IL-) 19 to acne vulgaris. This pilot study aims to look at difference in IL-19 serum concentration on degrees of severity of acne vulgaris.

**Methods:**

This is an analytical observational cross-sectional study. Sample subjects were patients with acne vulgaris who met the inclusion criteria. Enzyme-linked immunosorbent assay (ELISA) study was applied to measure IL-19 serum.

**Result:**

Analysis test found statistically significant difference between IL-19 serum concentration of group of patients with mild acne vulgaris and that of group of patients with severe acne vulgaris. Moreover, analysis revealed significant difference between IL-19 serum concentration of group of patients with moderate acne vulgaris and that of group of patients with severe acne vulgaris.

**Conclusions:**

There are differences in serum levels of IL-19 on the severity of acne vulgaris. The significant difference might show that inflammation has a core role in severity of acne vulgaris, and IL-19 might potentially be related to acne vulgaris.

## 1. Introduction

Acne vulgaris is a multifactorial disease which is associated with pilosebaceous follicle and results in inflammatory and noninflammatory lesions [1]. The pathogenesis of acne is attributed to increased sebum production, inflammatory processes, follicular hyperproliferation, and the proliferation of* Propionibacterium acnes (P. acnes)* [2]. Nowadays, it is stated that inflammation continuous to happen in early stage and late stage of acne vulgaris [3]; therefore, the inflammation does have a central role in the formation of both inflammatory and noninflammatory lesions in acne vulgaris [4].

The inflammation in acne vulgaris itself linked with* P. acnes*.* Propionibacterium acnes *stimulates keratinocytes through the Toll-like receptors (TLRs) to produce proinflammatory cytokines. An example of proinflammatory cytokines that are already known is interleukin- (IL-) 1*β* [5]. The other cytokines related to pathogenesis of acne vulgaris are IL-6, IL-8, IL-10, and IL-12 [6]. There is no study about IL-19 cytokines related to acne vulgaris or acne vulgaris severity.

Interleukin- (IL-) 19 is a cytokine expressed by epithelial cells with proinflammatory stimulation [7]. A distinctive feature of IL-19 is their ability of giving positive feedback loop to amplify themselves [8]; once they are activated in inflammatory process, they will continuously produce the cytokine. The present research sought to find out the difference in IL-19 serum concentration on degrees of severity of acne vulgaris; this is the pilot study of IL-19 serum in acne vulgaris patients.

The research belongs to an observational analytical study with cross-sectional study design. The target population includes acne vulgaris patients in Surakarta, while the accessible population involves acne vulgaris patients in Dr. Moewardi General Hospital of Surakarta and private specialist clinics which meet the inclusion criteria. Samples were taken using purposive sampling.

## 2. Method

The number of samples was determined using unpaired numerical analytical test. The inclusion criteria include subjects between the ages of 16 and 30 years clinically diagnosed with acne vulgaris who were willing to participate in the research and to fill in a questionnaire and a statement of willingness. Meanwhile, the exclusion criteria involve acne vulgaris patients undergoing systemic and topical treatment in the last two weeks and patients with other conditions related to an increase in IL-19 expression in serum or skin lesions such as psoriasis [9, 10], atopic dermatitis [10, 11], and asthma [12, 13].

To determine the degree of severity of acne vulgaris, anamnesis, clinical checkup, and picture taking were carried out. We used acne severity grading systems from Lehmann recommended in Indonesian acne expert meeting 2012 (Table 1) [14].

A 6 mL blood sample was drawn from every subject for an examination of IL-19 serum concentration. Such procedures which applied human IL-19 immunoassay with quantitative sandwich enzyme-linked immunosorbent assay (ELISA) (Quantikine ELISA Kits: R&D systems; ELISA reader 680 Bio-Rad) conform to the laboratory protocols. A number of 66 subjects comprising 3 groups (a group of patients with mild acne vulgaris, of patients with moderate acne vulgaris, and of patients with severe acne vulgaris) were obtained. The average ages of a group of patients with mild acne vulgaris, of patients with moderate acne vulgaris, and of patients with severe acne vulgaris are 21.55 ± 3.74 years, 22.18 ± 2.70 years, and 21.18 ± 3.90 years, respectively.

## 3. Results

**Figure 1:**
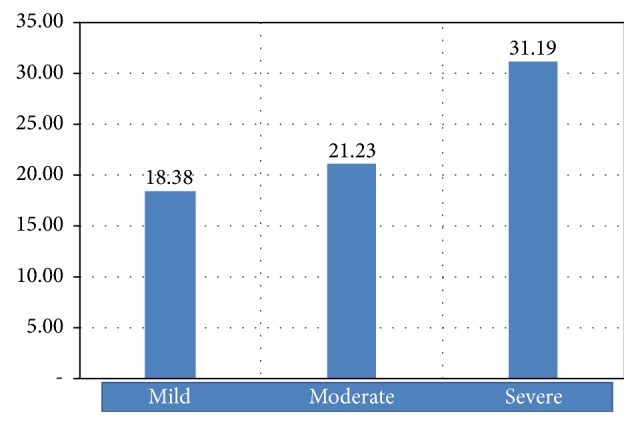
The difference in means of IL-19 serum (pg/ml).

The mean of IL-19 serum concentration of group of patients with mild acne vulgaris is 18.38 ± 9.59 pg/ml, that of group of patients with moderate acne vulgaris is 21.23 ± 11.99 pg/ml, and that of group of patients with severe acne vulgaris is 31.19 ± 20.36 pg/ml. The mean of IL-19 serum concentration of all groups is 23.60 ± 15.51 pg/ml.  Figure 1 presents the difference in means of IL-19 serum concentration among the groups.

Analysis of independent samples *t*-test using Mann–Whitney test found statistically significant difference between IL-19 serum concentration of group of patients with mild acne vulgaris and that of group of patients with severe acne vulgaris (*p* = 0.010) and no significant difference between IL-19 serum concentration of group of patients with mild acne vulgaris and that of group of patients with moderate acne vulgaris (*p* = 0.312). Moreover, analysis of dependent samples *t*-test revealed significant difference between IL-19 serum concentration of group of patients with moderate acne vulgaris and that of group of patients with severe acne vulgaris (*p* = 0.048) (Table 2).

## 4. Discussion

Acne vulgaris belongs to a complex and multifactorial disease of pilosebaceous unit. Acne pathogenesis cannot be viewed as impartial since its etiologies influence each other. Pathogenesis of acne vulgaris recently emphasizes inflammatory process. The latest finding indicates role of* P. acnes* as a triggering factor of inflammatory responses which exerts an influence on severity of acne vulgaris. Pathways underlying the formation of papules, pustules, and nodulocystic acne which involve cytokines were detected [15].* Propionibacterium acnes* proliferates in lipid-rich environment contribute to formation of inflammation, by inducing TLR-2 to release such proinflammatory cytokines as IL-1*β* [16]. The present research was intended to find out the difference in IL-19 serum concentration on various degrees of severity of acne vulgaris. The study from Kunz et al. stated that IL-1*β* could induce expression of IL-19 in keratinocytes both in vitro and in vivo [10]. Epithelial cells such as keratinocytes will produce IL-19 after being stimulated by IL-1*β* [17, 18]. It is found out that the release of proinflammatory cytokines of IL-1*β* may lead to the release of IL-19 despite the unknown special activation signal between IL-1*β* and IL-19 [11]. Such mechanism ends up with inflammatory processes and tissue damage in acne vulgaris patients and an increase in incidence and severity of acne vulgaris. Such cytokines are related and influence degrees of severity of acne vulgaris.

The research finding which signified the difference in IL-19 serum concentration on various degrees of severity of acne vulgaris is in line with a study conducted by Li et al., measuring IL-19 in patients with psoriasis, which emphasizes that, with reference to etiopathogenesis, the severity of both diseases is indicated by an increase in concentration of proinflammatory cytokines [19].

The measurement of IL-19 using serum was carried out by Li et al. to find out the correlation between IL-19 serum concentration and diabetic nephropathy. The measurement conducted on healthy control subjects revealed smaller IL-19 concentration (mean = 13.2 pg/ml) than on subjects with type 2 diabetes (mean = 40.5 pg/ml) [20], since IL-19 is only expressed in inflammatory location and condition. The present research detected proportional increase in IL-19 serum and degrees of severity of acne vulgaris. It may indicate inflammatory condition of acne vulgaris which can lead to an increase in IL-19, as detected in serum.

The measurement of expression of proinflammatory cytokines in acne vulgaris through serum was also carried out in several studies. Maulinda et al. examined IL-17 serum concentration on patients with papulopustular acne and comedonal acne with total subjects of 24 persons [21]. Another study was carried out by Liu et al. measuring IL-6 serum to predict effectivity of acupuncture for severe acne vulgaris [22].

Enzyme-Linked Immunosorbent Assay was applied in the research to measure IL-19 serum on research subjects. Enzyme-Linked Immunosorbent Assay refers to the most commonly used proteomic method with the best validity. The method enables us to detect antigens or cytokines through an antibody, while another antibody linked to an enzyme provides detection and an amplification factor. Due to accurate and sensitive detection of the antigen, ELISA has been considered the standard cytokine measurement method and is widely utilized in clinical laboratories and biomedical research [23].

The research results indicated the significant difference between moderate and severe acne vulgaris. The more the severe inflammation is, the bigger the detected IL-19 serum concentration will be. Such finding is in accordance with a research conducted by Yahil which shows that IL-19 production depends very much on the involved cells and expressing cell microenvironment, and therefore the more severe inflammation is, the higher expression of IL-19 will be [24].

## 5. Conclusion

There are differences in means of IL-19 serum concentration in acne vulgaris patients. The significant difference between IL-19 serum concentration of group of patients with moderate acne vulgaris and patients with severe acne vulgaris might show that inflammation has a core role in severity of acne vulgaris. This may lead to another research to find out that IL-19 might relate more to acne vulgaris inflammation and severity due to its unique features.

## Figures and Tables

**Table 1:** Grading classification Lehmann.

Grading	Comedones	Inflammatory lesions^*∗*^	Cyst	Total
Mild	<20	<15	-	<30
Moderate	20–100	15–50	<5	30-125
Severe	>100	>50	>5	>125

^*∗*^Inflammatory lesions: example, papule/pustule/nodules.

**Table 2:** Detecting the difference in means of IL-19 serum concentration among samples.

Detecting the difference in means	Mann–Whitney test	
	Value of *Z*-MW	*p*
Degrees of severity of acne vulgaris: mild-moderate	−1.011	0.312
Degrees of severity of acne vulgaris: mild-severe	−2.586	0.010^*∗*^
Degrees of severity of acne vulgaris: moderate-severe	−1.974	0.048^*∗*^

*Source*. Primary data 2017, processed; *Annotation*. ^*∗*^Significant at significance level of 5%.
